# Integrative approaches for the structure‐based functional understanding of the ethylene response in plants

**DOI:** 10.1111/tpj.70810

**Published:** 2026-03-24

**Authors:** Lisa Sophie Kersten, Raphael Josef Eberle, Georg Groth, Holger Gohlke

**Affiliations:** ^1^ Institute for Pharmaceutical and Medicinal Chemistry Heinrich‐Heine‐Universität Düsseldorf 40225 Düsseldorf Germany; ^2^ Institute of Biochemical Plant Physiology Heinrich‐Heine‐Universität Düsseldorf 40225 Düsseldorf Germany; ^3^ Institute of Bio‐ and Geosciences (IBG‐4: Bioinformatics), Forschungszentrum Jülich 52425 Jülich Germany

**Keywords:** ethylene signaling, ethylene receptor, ETR1, copper cofactor, copper chaperones, structural dynamics, protein–protein interactions, stress responses, post‐harvest spoilage, *Arabidopsis thaliana*

## Abstract

Ethylene is a key plant hormone regulating growth, development, and stress responses, yet the structural basis of its perception and signaling remains only partially understood. Ethylene receptors, which reside in the endomembrane network, act as dynamic signaling hubs that integrate hormone binding, copper (Cu^+^) cofactor coordination, and protein–protein interactions to control downstream pathways. Despite progress in characterizing individual domains, the full‐length structural organization of receptors and the mechanisms linking copper (Cu^+^) coordination to conformational signaling remain unclear. Equally, the functional significance of receptor multimerization and higher order clustering in shaping signaling robustness and cross‐talk is only beginning to emerge. To address these gaps, integrative approaches that combine structural biology, advanced spectroscopic techniques, targeted mutagenesis, molecular dynamics simulations, and molecular bioinformatics are employed. Recent advances in cryo‐electron microscopy (cryo‐EM), cross‐linking mass spectrometry, and super‐resolution imaging offer unprecedented opportunities to capture conformational states, map transient receptor interfaces, and visualize clustering dynamics in living cells. Complementary structure prediction tools together with hybrid quantum/classical simulations and perturbation analyses further connect local binding events to long‐range allosteric communication. This review focuses on these multidisciplinary strategies that pave the way toward a unified mechanistic framework of ethylene signaling.

## Significance Statement

Ethylene signaling governs key plant processes, yet how its receptors translate hormone binding and copper coordination into cellular responses remains unclear. Integrating structural biology, spectroscopy, and simulations reveals receptor organization and clustering, advancing a unified mechanistic framework for ethylene perception and signaling in plants.

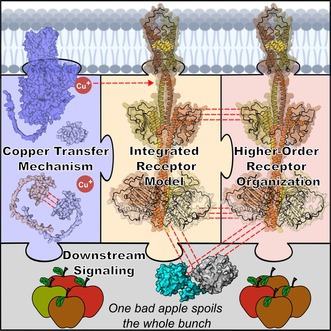

This review highlights how integrative structural, computational, and spectroscopic approaches are advancing our understanding of ethylene receptor architecture, dynamics, and signaling. These multidisciplinary strategies are uncovering how copper coordination, conformational transitions, and receptor clustering collectively shape ethylene perception and downstream responses.

## INTRODUCTION

Ethylene (C_2_H_4_) is the simplest alkene and is identified as a plant hormone. Despite its chemical simplicity, ethylene profoundly influences plant growth, development, and adaptation. It regulates key developmental processes, including seed germination, cell division, root hair formation, flower induction, fruit ripening, senescence, and organ abscission (Abeles et al., 1992; Burg & Burg, 1962; Corbineau et al., 2014; Dubois et al., 2018; Etchells et al., 2012; Feng et al., 2020; Liu et al., 2015; Mattoo, 1991; Wuriyanghan et al., 2009). Beyond development, ethylene plays a central role in plant responses to abiotic stresses such as drought, flooding, salinity, cold, and heat, as well as in attack by pathogens (Du et al., 2014; Guan et al., 2015; Perata, 2020; Tao et al., 2015; Thomashow, 2010; Zimmermann et al., 2009). Climate change further exacerbates the frequency and severity of these stress conditions, thereby potentially activating the negative effects of ethylene. Higher temperatures, for instance, increase ethylene biosynthesis and respiration rates, accelerating fruit spoilage and increasing susceptibility to post‐harvest stress. This not only reduces crop yield but also causes food to be lost across the supply chain due to premature aging and physiological disorders (Moretti et al., 2010). Given its multifaceted role, ethylene is a strategic entry point for dissecting plant signaling pathways. Its relevance in post‐germination development and post‐harvest spoilage underscores its value as a target for biotechnological applications aimed at improving crop resilience and shelf life.

Ethylene perception is mediated by a family of receptors localized at the endomembrane network (Dong et al., 2008). In *Arabidopsis thaliana*, five isoforms have been identified: ETR1 (ethylene response) (Schaller et al., 1995), ETR2 (Sakai et al., 1998), ERS1 (ethylene response sensor) (Hall et al., 2000), ERS2 (Hua et al., 1998), and EIN4 (ethylene insensitive) (Hua et al., 1998). These receptors act as negative regulators of ethylene signaling in plants (Hall et al., 1999). Despite their structural similarities, the functional roles and evolutionary significance of maintaining five distinct isoforms remain unclear.

**Figure 1:**
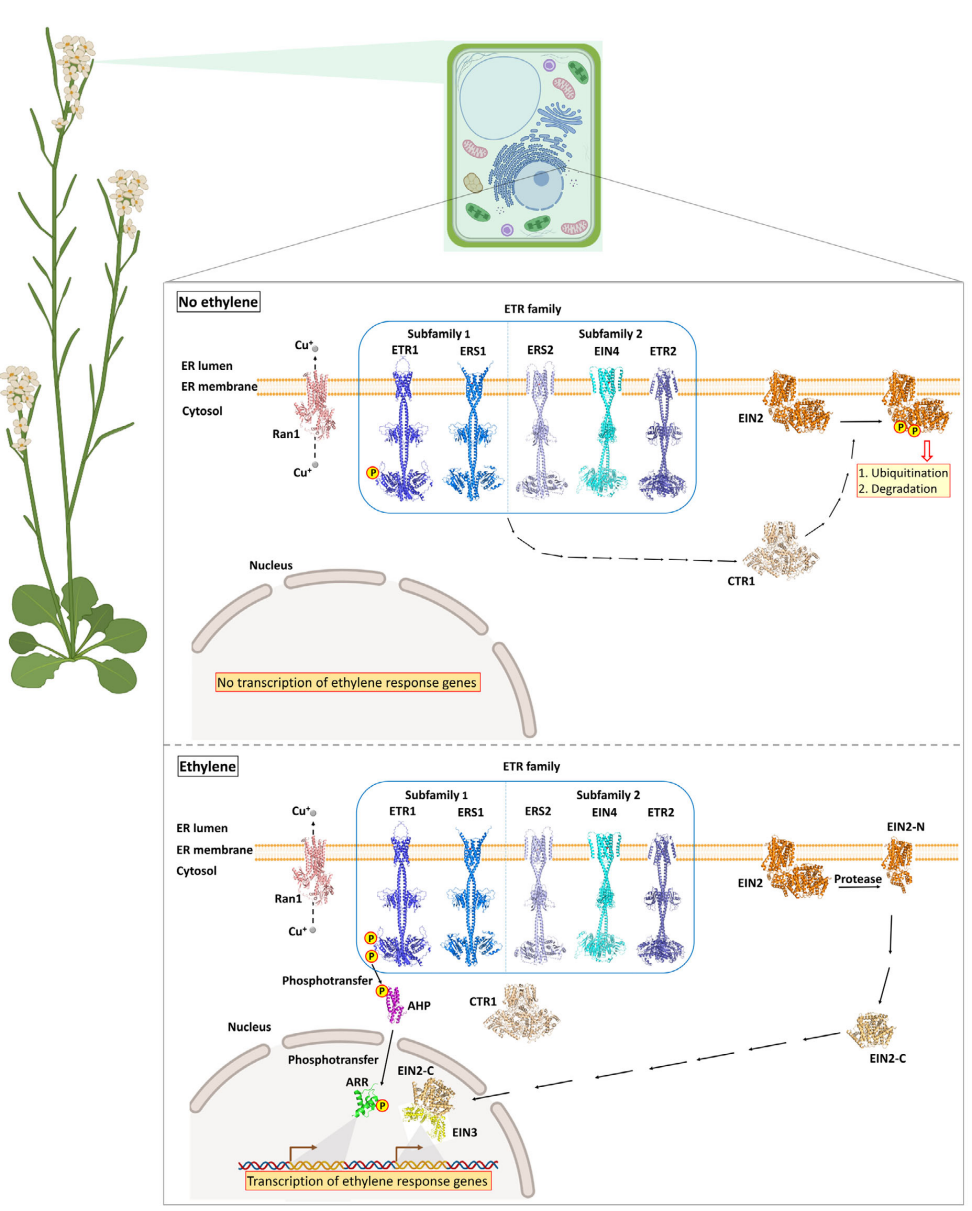
Model of the ethylene signaling pathway in *Arabidopsis thaliana*. The copper chaperone RAN1 transports copper ions (Cu^+^) to ETR1, enabling proper receptor biogenesis and high‐affinity ethylene binding (Dluhosch et al., 2025). In the absence of ethylene, ETR1 and other receptors activate CTR1, which phosphorylates the integral membrane protein EIN2 at its C‐terminal domain. Phosphorylated EIN2 is subsequently ubiquitinated and degraded. In the presence of ethylene, the hormone binds to the Cu^+^‐coordinated sensor domain of the receptors, causing CTR1 inactivation or sequestration. This results in reduced EIN2 phosphorylation, stabilization of the protein, and proteolytic cleavage by an unidentified protease. The released EIN2‐C fragment translocates into the nucleus, where it enhances EIN3/EIL1 transcriptional activity via interaction with ENAP1. Alternatively, ETR1 transfers a phosphoryl group from its histidine (DHp domain) to its RD, and subsequently to AHPs and ARRs, driving further transcriptional changes. The protein structures were predicted using AlphaFold3 (Jumper et al., 2021).

In the absence of ethylene, receptors actively suppress ethylene signaling by recruiting and activating the Raf‐like kinase CTR1 (constitutive triple response 1) (Clark et al., 1998). Activated CTR1 phosphorylates the C‐terminal domain of the integral membrane protein EIN2, thereby preventing it from transmitting the signal. EIN2 is further destabilized via ubiquitination mediated by F‐box proteins ETP1 and ETP2 (Chen et al., 2011; Ju et al., 2012; Qiao et al., 2012; Wen et al., 2012). Upon ethylene binding, receptor activity is inhibited, leading to CTR1 inactivation. EIN2 is stabilized and proteolytically cleaved, releasing its C‐terminal cytosolic fragment, which translocates into the nucleus. There, it promotes the accumulation and transcriptional activity of EIN3 and EIL1 (for ethylene insensitive3‐like), which in turn activate numerous ethylene response factors (ERFs) and downstream target genes (Figure 1) (Ju et al., 2012; Qiao et al., 2012; Wen et al., 2012). This cascade constitutes the canonical ethylene signaling pathway. Additionally, recent evidence supports the existence of an alternative, non‐canonical phosphorelay pathway. Here, ETR1 transfers a phosphoryl group from its dimerization and histidine phosphotransfer (DHp) domain to its own receiver domain (RD), and subsequently to AHPs (*Arabidopsis thaliana* histidine phosphotransfer proteins), culminating in phosphorylation of ARRs (Type‐A Arabidopsis response regulators), thereby modulating ethylene response (Binder, 2020; Binder et al., 2004, 2018; Mira‐Rodado et al., 2012; Nemhauser et al., 2006; Street et al., 2015; Zdarska et al., 2019; Zhao et al., 2020).

Among the *Arabidopsis* receptors, ETR1 is the most extensively studied. It binds ethylene with high affinity via an essential Cu^+^ cofactor located in the transmembrane sensor domain (TMD), enabling plants to respond to a wide range of ethylene concentrations (O'Malley et al., 2005). Mutational analyses have identified conserved residues in the TMD that are critical for high‐affinity binding and for coupling ligand perception to conformational changes in the receptor complex.

The activity of ETR1 can be modulated by a variety of small molecules. Notably, ethylene is the only endogenous, physiologically relevant ETR1 ligand *in vivo*. Most other described small‐molecule modulators are applied exogenously and primarily serve as experimental probes or tools for post‐harvest/agricultural applications, rather than functioning as true endogenous regulators. Ethylene itself acts as an inverse agonist of ETR1, while carbon monoxide and isocyanides also trigger ethylene responses in plants (Bisson & Groth, 2012; Burg & Burg, 1967; Sisler, 1977). On the other hand, several strained alkenes function as ETR1 antagonists, blocking ethylene signaling. The best known one is 1‐methylcyclopropene (1‐MCP), widely used in agriculture to block ethylene perception and thereby delay fruit ripening and senescence during post‐harvest storage (Sisler et al., 1996). Another potent antagonist is the *R*‐enantiomer of *trans*‐cyclooctene. The corresponding *S*‐enantiomer is a weaker antagonist, consistent with a chiral binding site (Pirrung et al., 2008; Sisler et al., 1990). In addition to small molecules, peptides that modulate ETR1 signaling have been identified, providing further regulatory approaches (Bisson et al., 2016; Kessenbrock et al., 2017; Milic et al., 2018; Mudge et al., 2025).

Due to their ability to block ethylene signaling, ETR1 antagonists are highly relevant to post‐harvest and crop management strategies. Yet, despite decades of research, important questions remain open. The structural basis of high‐affinity ligand binding is still incompletely understood, though recent studies have highlighted the roles of conserved histidine, cysteine, and aspartate residues in the TMD (Azhar et al., 2023). Similarly, the conformational changes that propagate from the Cu^+^ cofactor in the TMD to the cytoplasmic kinase domains, CTR1, and ultimately, EIN2, remain unclear. Additionally, the activity of ETR1 is dynamically regulated by receptor multimerization, interactions with accessory proteins, and modulation by regulatory peptides. These factors provide additional layers of control, integrating ethylene perception with broader hormonal and stress‐response networks.

Given the fundamental and translational relevance of ethylene signaling – from seedling development to post‐harvest preservation of fruits and vegetables – elucidating receptor structure and function remains a high priority. This review highlights recent advances in the structural and functional understanding of ethylene receptor ETR1 and discusses how multifaceted integrative approaches integrating biochemical, biophysical, and cellular methodologies with structural studies and molecular modeling are beginning to bridge the gap between molecular events at the receptor and physiological responses at the whole‐plant level.

## THE MODULAR CONSTRUCTION OF THE ETHYLENE RECEPTOR FAMILY

Ethylene receptors are modular proteins composed of two principal regions: a TMD, embedded in the ER membrane and responsible for ethylene binding and receptor localization, and a cytosolic multidomain region responsible for signal transduction and protein–protein interactions.

Subfamily I receptors (ETR1 and ERS1) consist of three membrane integral helices (TMH1‐3), whereas subfamily II receptors (ETR2, ERS2, and EIN4) possess an additional fourth TMH. Within these helices, a conserved set of cysteine and histidine residues is implicated in chelating the Cu^+^ cofactor (Cutsail et al., 2022; Rodriguez et al., 1999; Schaller et al., 1995), which in turn selectively and non‐covalently binds the ethylene molecule.

The cytosolic part of ETR1 displays three domains characteristic of hybrid‐type histidine kinase receptors: the GAF (cGMP‐specific phosphodiesterases, adenylyl cyclases, and FhlA) domain implicated in receptor interactions (Gao et al., 2008; Grefen et al., 2008), the histidine kinase (HK) consisting of a dimerization (DHp) and a catalytic (CA) domain, and finally the receiver domain (RD) mediating downstream signaling (Chang et al., 1993). Functionally, ethylene receptors act as negative regulators of the signaling pathway, such that the receptors are “ON” in the absence of the plant hormone, actively suppressing the ethylene response, and “OFF” when bound to the plant hormone, allowing for de‐repression of the ethylene response (Binder, 2020; Hua & Meyerowitz, 1998; Wang et al., 2006).

The following subsections will briefly review recent advances in our understanding of the TMD, with emphasis on its roles in ligand perception and Cu^+^ coordination, followed by a discussion of the cytosolic multidomain region and its contributions to receptor interactions and downstream signaling dynamics.

### The transmembrane sensor domain: copper coordination and ligand perception

The TMD of ETR1 is responsible for binding the plant hormone ethylene and initiating downstream signaling. Despite its central role, the structure, mechanism of action, and Cu^+^ stoichiometry of the TMD have remained elusive for decades, limiting our understanding of how hormone perception is transduced into a biochemical signal.

Despite considerable progress, an experimentally resolved high‐resolution structure of the ETR1 TMD is still lacking (Rüffer et al., 2024). To address this gap, an *ab initio* approach combined with evolutionary information was employed in 2019 to generate the first structural model of the TMD (Schott‐Verdugo et al., 2019). The obtained model was refined using experimental data on Cu^+^ stoichiometry and validated through mutagenesis studies. Molecular dynamics simulations further illustrated how ethylene could bind in proximity to the Cu^+^‐binding sites. These integrative approaches provided the first framework for how ethylene might interact with the receptor, revealing a dimeric arrangement stabilized by Cu^+^ cofactors.

Alternative structural views soon emerged with the advent of AlphaFold (Jumper et al., 2021), which predicted a distinct helix arrangement and proposed different dimerization interfaces and Cu^+^‐binding geometries. To experimentally discriminate between these models, site‐directed spin labeling combined with electron paramagnetic resonance (EPR) spectroscopy was applied to liposome‐reconstituted receptors. The resulting distance restraints aligned more closely with the *ab initio* model than with the AlphaFold prediction, although neither model fully recapitulated the experimental data (Kugele et al., 2022). These discrepancies underscore the dynamic and conformationally flexible nature of the TMD and highlight the challenges of resolving its precise structural organization.

Complementary studies in tomato receptors (e.g., LeETR2) offer additional comparative insights. While NMR‐based structural analysis was hampered by the hydrophobicity of the TMDs, fusion constructs of LeETR2 yielded analyzable spectra in organic solvents. Notably, AlphaFold2 predictions produced highly convergent dimer models, and docking combined with molecular dynamics simulations identified potential ethylene‐binding sites within the TMD (Wei et al., 2022). Although limited in resolution, these findings support the existence of conserved structural motifs across species, reinforcing the broader relevance of the TMD architecture in ethylene receptor function.

Central to ethylene binding is a Cu^+^ cofactor (Rodriguez et al., 1999) coordinated by the TMD. Spectroscopic analyses, including X‐ray absorption and extended X‐ray absorption fine structure (EXAFS) analyses, revealed a low‐coordinate Cu^+^‐binding site with a mixed nitrogen/oxygen and sulfur ligand environment. These studies corroborated the involvement of Cysteine 65 and Histidine 69 (located in TMH 2) as key Cu^+^‐coordinating residues. Both are highly conserved across ethylene receptor homologs and are essential for receptor function. Importantly, both EXAFS data and quantum mechanics/molecular mechanics umbrella sampling simulations demonstrated that Cu^+^ coordination is dynamically modulated upon ethylene binding. Specifically, the Histidine 69‐Cu^+^ distance increases in the ligand‐bound state, suggesting that rearrangement of the Cu^+^ coordination sphere serves as a molecular trigger for signal initiation (Cutsail et al., 2022).

Recent genetic and structural studies have contributed to this model by implicating a conserved aspartate residue within the TMD as a critical determinant of ethylene‐binding affinity. Substitution of this residue to asparagine results in a functional receptor with approximately 100‐fold reduced binding affinity (Aman et al., 2025). Importantly, this residue is highly conserved across plant and bacterial ethylene receptor‐like proteins, although natural variants exist, highlighting its relevance for species‐specific tuning of ethylene‐sensitivity (Azhar et al., 2023; Wang et al., 2006). Structural analysis based on the AlphaFold model suggests that the aspartate not only contributes directly to ligand binding but also forms a salt bridge to a conserved lysine residue, providing a plausible mechanism for coupling ligand binding to conformational signaling. These findings point to mechanistic parallels with mammalian olfactory receptors, underscoring the broader significance of the ethylene receptor family (Aman et al., 2025; Azhar et al., 2023).

Beyond intrinsic structural determinants, extrinsic factors also modulate receptor activity at the TMD level. A notable example is the short (36 amino acids) *Arabidopsis* peptide POLARIS (PLS), which binds Cu^+^ in a thiol‐dependent manner and interacts directly with the ETR1 TMD. PLS‐ETR1 interactions are enhanced in the presence of Cu^+^ and provide a Cu^+^‐dependent mechanism for repression of ethylene signaling. Intriguingly, PLS transcription is upregulated by auxin and downregulated by ethylene, linking receptor activity to hormonal cross‐talk and positioning the TMD, in addition to its role as ligand binding region, as a regulatory hub for signal integration (Mudge et al., 2025).

Taken together, current research establishes the TMD as a dynamic and multifunctional sensor module. It directly couples high‐affinity ethylene binding via Cu^+^ coordination and conserved residues to conformational changes that initiate signaling, while also serving as a platform for modulation by ETR1 antagonists, regulatory peptides, and potentially other interacting factors. Yet, several key questions remain unanswered:

- What is the native structural arrangement of the TMD?
- How are conformational changes in the Cu^+^‐binding site propagated across transmembrane helices?
- How does the TMD communicate with the cytoplasmic signaling domains?

Addressing these questions will require integrative methodologies combining spectroscopy, mutational analyses, and high‐resolution structural techniques such as cryo‐EM, alongside molecular modeling and simulations, to capture the conformational landscape of this unique receptor family.

### The cytosolic multidomain: highly flexible and signal‐forwarding core of the receptor

Functional studies on each isolated cytosolic domain provided key insights into their structural integrity and signaling roles. The GAF domain has been shown to mediate peptide‐binding (e.g., NOP1) and interact with EIN2 (Bisson & Groth, 2010; Milic et al., 2018). Activity, folding, and dimerization of the kinase domain were demonstrated by phosphorylation assays (Berleth et al., 2019; Voet‐Van‐Vormizeele & Groth, 2008), while the RD's function was validated by AHP1 binding in fluorescence polarization (FP) assays (Scharein et al., 2008). Collectively, these findings, supported by structural studies that will be discussed in the subsequent section, establish the ETR1 cytosolic multidomain as a highly flexible signaling module, likely essential for dynamic ethylene signal processing and transmission.

The crystal structure of the ETR1 RD (Residues 605–738; PDB code: 1DCF) reveals a canonical fold typical of bacterial RD in two‐component systems, which is characterized by a five‐stranded β‐sheet core surrounded by five α‐helices in a (βα)_5_ arrangement, with the active site for phosphorylation located on the β‐sheet. Phosphorylation of a conserved aspartate residue in this domain induces a conformational change, leading to the activation of the associated output domain, to generate a physiological response (Bourret, 2010). The RD forms a homodimer in solution and in crystals, with dimerization mediated by the C‐terminal region, forming an extended β sheet with the partner strand. Comparative analysis with bacterial RD structures suggests that phosphorylation‐dependent dimerization and monomerization may regulate activity. Notably, the active site architecture of the ETR1 RD diverges from that of bacterial Mg^2+^‐bound RDs (Bellsolell et al., 1994; Stock et al., 1993). The γ‐loop positioned immediately after the phosphorylatable aspartate typically primes the site for phosphorylation. In contrast, in the metal‐free ETR1 RD, this loop adopts a conformation incompatible with canonical phosphorylation, unless major structural rearrangements occur (Müller‐Dieckmann et al., 1999). Further insights were provided by NMR studies (Hung et al., 2016), which confirmed the unusual orientation of the γ‐loop observed in the crystal structure. This conformation renders the domain structurally unfavorable for phosphorylation. Based on these findings, ETR1 RD was classified as an atypical response regulator, likely functioning in a constitutively active state that transmits signals to downstream factors via protein–protein interactions rather than by phosphorylation‐dependent switching (Hung et al., 2016).

The ETR1 histidine kinase consists of the dimerization domain (DHp) and the catalytic domain (CA). Structural insights into the DHp domain were obtained from its homolog ERS1, which shares 84% sequence identity with ETR1. The ERS1 DHp domain was crystallized and resolved to 1.90 Å resolution (Residues 308–407; PDB code: 4MTX) (Mayerhofer et al., 2015). The structure adopts a canonical hairpin configuration formed by two antiparallel helices connected via a short loop, consistent with the typical fold described for prokaryotic homologs (Casino et al., 2009; Mayerhofer et al., 2015). Within this domain, histidine 353, the conserved phospho‐acceptor site, is solvent‐exposed and exhibits conformational flexibility, suggesting dynamic accessibility during autophosphorylation (Mayerhofer et al., 2015). The CA domain of ETR1 was also crystallized and resolved to 1.90 Å resolution (Residues 407–589; PDB code: 4PL9). It displays a characteristic α/β‐sandwich fold, structurally closely related to bacterial CA domains (Casino et al., 2009; Mayerhofer et al., 2015). One layer of the sandwich fold consists of a mixed five‐stranded β‐sheet (βB and βD–βG), while the opposing layer consists of three helices (α3–α5) and a pair of short antiparallel β‐strands (βA and βC). The CA domain was crystallized in complex with ADP, which binds to a well‐defined nucleotide‐binding pocket. The purine moiety of ADP binds to a hydrophobic cavity formed by phenylalanine 474, isoleucine 518, and isoleucine 526, with phenylalanine 474 also interacting with lysine 473, placing ETR1 within the canonical class of histidine kinases. Key hydrogen bonding interactions involve aspartate 513 with the adenine base and lysine 529 with the ribose O2′. Notably, successful crystallization of the CA domain required CdSO_4_, as attempts without nucleotide/CdSO_4_ or with Mg^2+^/Mn^2+^ failed, suggesting these ligands are essential for stabilizing flexible regions of the protein. The final structure revealed 11 Cd^2+^ sites per monomer with eight located at surface‐exposed side chains and three mediating protein–ADP interactions, supporting their structural role. In the nucleotide‐binding pocket, a Cd^2+^ ion replaced the usual water‐mediated contact between a conversed aspartate residue and the ADP. This cation is coordinated by a nearby cysteine and an anion. Although the biological identity of this cation is unclear, the coordination geometry and available space strongly suggest that this position in ETR1 acts as a cation‐binding site. Sequence comparisons suggest that this site is likely restricted to subfamily 1 and absent from subfamily 2 members (Mayerhofer et al., 2015).

## FULL‐LENGTH ETR1: MEMBRANE INTEGRATION, COFACTOR LOADING, AND SIGNALING INTERFACES

### Reconstitution and receptor flexibility

The ethylene receptor family consists of membrane‐bound protein kinases that integrate hormone perception with intracellular signaling. Historically, investigations into their biochemical, biophysical, structural, and dynamic properties have relied on detergent‐solubilized preparations, which often fail to preserve native organization. To overcome these limitations, Lemke et al. (2023) developed a protocol for reconstituting full‐length ETR1 into lipid nanodiscs (NDs) – discoidal lipid bilayers stabilized by amphipathic membrane scaffold proteins (MSPs). This approach enabled the incorporation of full‐length *Arabidopsis thaliana* ethylene receptor ETR1 into a near native membrane environment. Size exclusion chromatography (SEC), small‐angle X‐ray scattering (SEC‐SAXS), and solution nuclear magnetic resonance (NMR) confirmed that ETR1 retained its native dimeric conformation within the nanodisc assembly. Subsequent NMR analyses revealed that the N‐terminal TMD is stably embedded within the lipid bilayer, preserving its structural integrity. In contrast, the cytosolic domains exhibit pronounced conformational flexibility in the apo state. This dynamic behavior is primarily attributed to interdomain linker regions between the GAF, HK, and RDs, which likely facilitate domain rearrangement during signal transmission (Lemke et al., 2025).

### Cytosolic domain organization

Truncated receptor constructs have been instrumental in elucidating the architecture of the cytosolic domains. SAXS analyses of ETR1‐ΔTMD (residues 158–738) revealed a dimeric assembly characterized by a central dimerization (DHp) stalk flanked by peripheral catalytic (CA) and receiver (RD) domains (Mayerhofer et al., 2015). The overall topology resembles a dumbbell‐shaped structure, with the GAF domain positioned at one end and the CA/RD modules at the opposite pole. Flexible interdomain linkers connect these modules and can adopt more compact conformations in the presence of stabilizing agents such as ADP or divalent cations, suggesting a dynamic structural landscape responsive to ligand binding. Complementary NMR studies revealed that the ETR1 RD exhibits atypical rigidity, lacking the conformational plasticity typically observed in phosphorylatable RDs even under conditions that mimic phosphorylation, such as Mg^2+^ and ${\mathrm{BeF}}_{3}^{-}$ treatment (Hung et al., 2016). This structural rigidity may underlie the limited phosphorylation activity of ETR1 compared with canonical histidine kinases and supports its classification as an atypical RD.

### Metal‐binding sites within ethylene receptors

High‐affinity ethylene binding is mediated by a Cu^+^ cofactor coordinated by conserved cysteine and histidine residues within the TMD (Cutsail et al., 2022; Rodriguez et al., 1999). This Cu^+^ ion not only facilitates selective, non‐covalent ethylene binding (Rodriguez et al., 1999) but also contributes to dimer stabilization (Lemke et al., 2025). Comparative metal substitution studies revealed that Au^+^ ions can functionally replace Cu^+^ and support ethylene perception, whereas Ag^+^ ions act as antagonists, blocking ethylene responses in plants (Binder et al., 2007). A second metal‐binding site resides within the nucleotide‐binding pocket of the CA domain, where Mn^2+^, Mg^2+^, Ca^2+^, or Cd^2+^ ions are required for ATP binding and kinase activity (Gamble et al., 1998; Lemke et al., 2025). While no additional metal‐binding sites have been definitely identified, it remains plausible that metal coordination contributes to conformational stabilization or modulates dynamic regions of the receptor.

### Cofactor delivery by copper chaperones

Because free copper ions are highly cytotoxic to the cell due to their ability to catalyze ROS formation, plants employ specialized copper chaperones to ensure targeted and safe intracellular delivery (Pufahl et al., 1997). Central to this process are members of the ATX1 family of soluble copper chaperones, which shuttle Cu^+^ across the cytosol to the P‐type ATPase RAN1 and ultimately to ETR1, thereby maintaining tight control of intracellular copper homeostasis (Hoppen et al., 2019).

In plants, the ATX1 family comprises not only classical ATX1 homologs but also the related CCH chaperone, featuring an extended C‐terminal tail (Pufahl et al., 1997). Biophysical analyses indicate that this extension is intrinsically disordered and its deletion reduces the chaperone's tendency to dimerize, suggesting a role in modulating oligomeric state and possibly the interaction specificity. Computational studies further support this hypothesis, showing that the C‐terminal extension provides transient contacts that reinforce the overall dimer structure (Dluhosch et al., 2024).

Cu^+^ transfer of ethylene receptors also involves RAN1, a transmembrane P‐type ATPase embedded in the ER membrane (Hirayama et al., 1999). Recent biochemical and computational work has provided direct evidence for protein–protein interactions among RAN1, ATX1‐like chaperones, and ETR1. These findings support a stepwise Cu^+^ transfer mechanism, in which the soluble chaperones deliver Cu^+^ from the plasma membrane to RAN1, which then transfers the Cu^+^ cofactor to ETR1. Although the precise molecular mechanism of this transfer has not been fully elucidated, domain‐level analyses of complex formation suggest that specific copper‐binding motifs (CBMs) in both the chaperones and RAN1 interact with each other and coordinate the transient protein–protein interactions required for efficient Cu^+^ delivery. Understanding these pathways is key to deciphering how ethylene receptors acquire their essential Cu^+^ cofactor, which is indispensable for ligand binding and receptor functionality (Dluhosch et al., 2025).

### Downstream signaling via the Raf‐like Ser/Thr kinase CTR1

Ethylene signal transduction relies on receptor‐associated proteins that couple hormone perception to transcriptional reprogramming in the nucleus. In the canonical *Arabidopsis thaliana* pathway, the Raf‐like Ser/Thr kinase CTR1, consisting of an N‐terminal regulatory domain and a C‐terminal kinase domain, functions as a key negative regulator downstream of ethylene receptors. In the absence of ethylene, receptor‐mediated activation of CTR1 maintains EIN2 in a phosphorylated state, causing degradation of EIN2 and thus preventing signal transmission. Ethylene binding to the receptors induces conformational changes that inactivate CTR1, allowing EIN2 to be stabilized and cleaved to initiate downstream transcriptional responses (Clark et al., 1998). While the overarching framework of this pathway is established, the detailed molecular mechanism by which conformational changes in the ethylene receptors are transmitted to CTR1 remains unresolved. Future structural studies of intact ethylene receptor‐CTR1 complexes will be essential to elucidate this mechanism.

### Ethylene receptor multimerization and cooperative signaling

A distinctive feature of ethylene signaling is the presence of multiple receptor isoforms within the plant genomes. *Arabidopsis* encodes five receptors classified into two subfamilies (Hall et al., 2000; Hua et al., 1998; Sakai et al., 1998; Schaller et al., 1995). Although they share core features, they differ in expression profiles and biochemical properties (Grefen et al., 2008; Hall et al., 2012; Wang et al., 2003). The functional implications of this multiplicity remain an active area of investigation (Binder, 2008; Gallie, 2015). A compelling hypothesis is that isoform diversity allows for ethylene perception across a broad concentration range while allowing tissue‐ and developmental stage‐specific tuning of sensitivity. Additionally, recent studies revealed that ethylene receptors do not act as isolated units. Instead, they assemble into higher order oligomers through homomeric and heteromeric interactions. Specifically, the GAF and RDs of ethylene receptors have been implicated as key mediators of these associations, suggesting that specific interdomain contacts facilitate receptor clustering (Bisson & Groth, 2010; Chen et al., 2010; Gao et al., 2008). Although the multimerization of ethylene receptors requires further investigation, it is hypothesized that receptor clustering may enhance ligand sensitivity and buffer signaling noise. This process entails the coupling of adjacent receptor dimers to average out stochastic binding events while simultaneously amplifying genuine ligand‐induced signals through cooperative inter‐dimer communication. Furthermore, receptor clustering could coordinate downstream responses across tissues.

## INTEGRATION AND CONCEPTUAL MODELS

Ethylene signaling via ETR1 and related receptors emerges from a multilayered interplay of ligand binding, metal coordination, conformational changes, and higher order receptor assembly within the ER membrane. Together, these processes position the receptors as dynamic hubs that connect local biochemical events to global signaling outcomes.

At the molecular level, Cu^+^ coordination within the TMD provides the basis for high‐affinity ligand binding, while conformational changes propagate the signal to the cytosolic domains. Signal output is further shaped by receptor–CTR1 interactions, and receptor multimerization introduces an additional layer of regulation that modulates sensitivity, robustness, and cooperative responses.

While domain‐specific studies have revealed many details, the challenge remains to integrate these findings into a coherent mechanistic picture. Achieving this requires bridging scales– from electronic rearrangements at the Cu^+^‐binding site to dynamic allostery across domains, and from membrane‐level receptor oligomerization to transcriptional reprogramming in the nucleus. Addressing these questions will require multidisciplinary approaches that integrate structural biology, cell biology, and computational modeling, ultimately linking receptor biogenesis and signaling to plant development and stress adaptation.

### Cofactor delivery as a biogenesis checkpoint

High‐affinity ethylene binding requires precise insertion of a Cu^+^ cofactor into the TMD of ethylene receptors. This process is orchestrated by the soluble chaperones ATX1 and CCH in concert with the P‐type ATPase RAN1. While the key players and their interaction interfaces are well characterized, specific amino acid contacts that stabilize or destabilize these transient complexes remain unresolved. Given that non‐metalated receptors are inactive, while properly metalated receptors gain full signaling competence, resolving these molecular interactions would provide crucial opportunities to selectively modulate ethylene receptor activity.

To dissect these contacts, molecular dynamics (MD) simulations of the chaperone‐RAN1‐ethylene receptor complexes can be followed by MM‐PBSA or MM‐GBSA free energy calculations to identify hot spot residues (Gohlke et al., 2003), crucial for protein–protein interactions. These computational predictions can be experimentally validated by site‐directed mutagenesis, with binding affinities quantified via isothermal titration calorimetry (ITC), microscale thermophoresis (MST), or surface plasmon resonance (SPR). Cross‐linking mass spectrometry (XL‐MS) can provide additional spatial restraints on residue proximities within transient complexes, while NMR chemical shift perturbation of isolated domains may reveal interface residues involved in metal transfer.

Beyond pairwise interactions, co‐evolutionary analyses across plant species could highlight conserved residues that mediate Cu^+^ handover, whereas in planta complementation assays using mutant variants of ATX1, CCH, or RAN1 would establish the physiological importance of these hot spots. Taken together, combining computational mapping with biochemical and genetic validation offers a powerful strategy to resolve how Cu^+^ delivery is choreographed at the atomic level and to define the molecular checkpoints that determine receptor activation.

### From ligand binding to cytoplasmic signaling

Spectroscopic analyses combined with hybrid quantum/classical simulations have revealed that ethylene binding induces subtle rearrangements in the Cu^+^ coordination sphere within the ETR1 TMD. However, it remains elusive how these local changes are propagated across the TMD to the cytosolic domains and ultimately influence downstream targets, such as CTR1. Addressing this question requires an allosteric perspective: ligand binding at the Cu^+^‐binding site must be understood not only as a local coordination event, but also as a trigger that reshapes the conformation and dynamics of the entire receptor.

To unravel this process, a combination of computational and experimental methods will be essential. Quantum mechanical approaches are particularly suited and required to capture the electronic and structural rearrangements within the Cu^+^‐binding site upon ligand binding. These insights can then be embedded into larger‐scale all‐atom MD simulations, which, when coupled with rigidity theory‐based perturbation analyses (Pfleger et al., 2017), can map pathways of signal transduction from the TMD to the cytosolic domains. On the experimental side, FRET‐based sensors and SAXS analyses of engineered receptor variants can provide restraints for conformational ensembles and validate predicted motions. Ultimately, high‐resolution methods such as cryo‐EM of full‐length receptors or receptor–CTR1 complexes, together with cross‐linking mass spectrometry, hold the promise of directly visualizing how ligand binding reshapes receptor dynamics, modulates activity, and alters interactions with downstream partners.

### Receptor clustering and cooperative signaling

An additional challenge in ethylene signaling is the coexistence of five receptor isoforms in *Arabidopsis thaliana*. While all isoforms act as negative regulators, their specializations are unclear. An important open question is why plants have maintained multiple receptor subtypes and how these isoforms contribute to signaling robustness.

One possible explanation is their ability to assemble into higher order complexes at the ER membrane. This clustering is thought to expand the dynamic range of perception, enable cooperative signaling, and enhance signal robustness. Yet, the molecular determinants of these interactions, particularly the specific amino acid residues that stabilize multimeric assemblies, remain to be resolved.

To dissect these interfaces, structural modeling of the GAF and RDs can be combined with MD simulations and MM‐PB/GBSA analyses to identify hotspot residues critical for dimerization and cluster stabilization. Targeted mutagenesis of these residues, followed by quantitative assays of receptor–receptor interactions (e.g., BiFC, split‐ubiquitin, or co‐purification assays), would provide direct validation. Cross‐linking strategies using cysteine substitutions at predicted hot spots can further probe the stability and orientation of receptor complexes.

At the same time, higher order clustering must be understood in its native cellular environment. Cross‐linking mass spectrometry of ER‐enriched extracts can reveal proximities between receptors in planta, while super‐resolution imaging (STED, PALM/STORM) of fluorescently tagged receptors can visualize cluster organization and dynamics. On the computational side, coarse‐grained MD simulations allow exploration of how multiple receptors self‐organize into arrays and how such assemblies could amplify weak ligand‐binding events into robust signaling outputs.

## PERSPECTIVES AND FUTURE OUTLOOK

**Figure 2:**
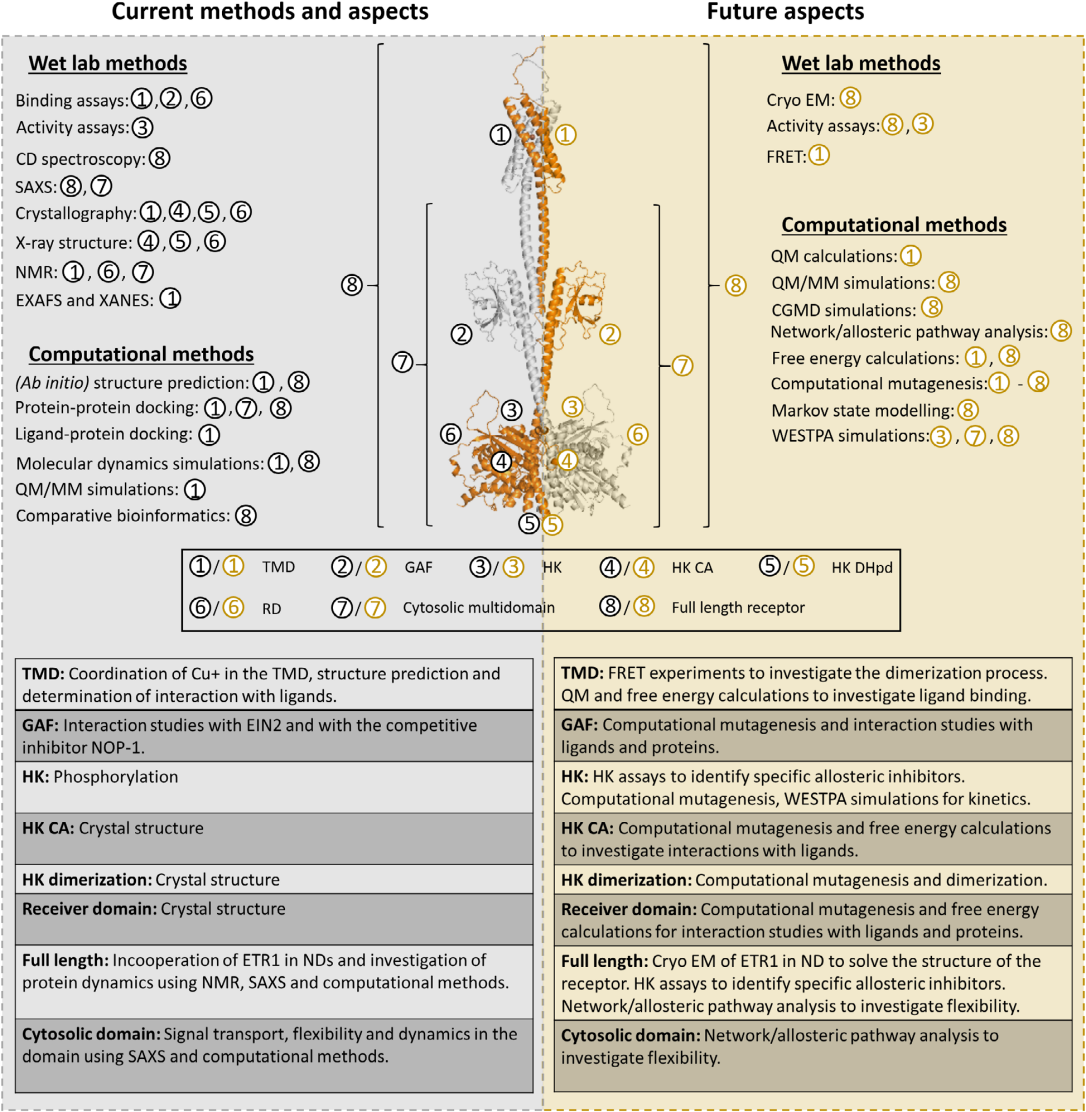
Summary of current and future methods and aspects to investigate ETR1. The ETR1 subdomains are labeled, and the corresponding aspects and scientific questions behind them are explained. The approaches include quantum chemical (QM) and hybrid quantum chemical/molecular mechanics (QM/MM) calculations to investigate ligand binding; coarse‐grained molecular dynamics (CGMD) to study receptor clustering and higher order assembly; and advanced sampling methods, such as the Weighted Ensemble Simulation Toolkit with Parallelization and Analysis (WESTPA), which capture rare events and long‐timescale transitions.

Despite substantial progress in elucidating the ethylene pathway (Figure 2, Box 1), fundamental questions remain (Box 2).

Box 1 **Bullet point summary**

- Ethylene receptors, with ETR1 as the prototype, integrate a transmembrane sensor domain (TMD) with a cytosolic multidomain region responsible for downstream signaling.
- High‐affinity ethylene binding requires a copper (Cu^+^) cofactor, which is coordinated by conserved cysteine and histidine residues within the TMD.
- Receptor biogenesis requires copper (Cu^+^) delivery by ATX1/CCH chaperones and the P‐type ATPase RAN1, forming a critical checkpoint for functional activation.
- ETR1 cytosolic domains (GAF, HK, RD) exhibit high conformational flexibility, enabling dynamic protein–protein interactions, regulation of CTR1, and integration into higher‐order signaling networks.
- Receptors assemble as multimeric complexes within the ER membrane, coordinating ligand binding, cofactor incorporation, and receptor clustering to modulate signaling outputs.

Box 2 **Open questions**

- What is the high‐resolution structure of full‐length ethylene receptors, and how are ligand‐induced conformational changes transmitted from the TMD to cytosolic domains?
- How does ethylene binding to the copper (Cu^+^) cofactor in the TMD initiate structural rearrangements that result in CTR1 inactivation?
- How do small peptides, antagonists, or alternative ligands modulate receptor activity at the structural and functional level?
- What is the molecular mechanism underlying receptor multimerization, and how does oligomeric clustering affect signaling robustness, dynamic range, and cross‐talk with other hormonal or stress‐response pathways?
- Can integrative structural and functional approaches pave the way for targeted modulation of receptor activity to enhance crop resilience and post‐harvest performance?

The precise structural organization of full‐length ethylene receptors is still unresolved, and it remains unclear how Cu^+^ coordination within the TMD drives conformational changes that are transmitted to the cytosolic signaling domains and ultimately modulate CTR1 and EIN2 activity. Likewise, the importance of the five receptor isoforms, as well as the structural and functional significance of receptor multimerization and higher order clustering, is beginning to be appreciated.

Nevertheless, recent advances have provided important footholds. Integrative approaches combining spectroscopy, mutagenesis, molecular dynamics simulations, and structural modeling have begun to reveal how ethylene perception is coupled to Cu^+^ coordination and conformational signaling. Concurrent research on cytosolic domains and full‐length receptors in membrane‐mimetic systems has emphasized the remarkable flexibility and modularity underlying receptor function. Together, these findings support a model in which ethylene receptors act as dynamic signaling hubs integrating hormone binding, Cu^+^ cofactor delivery, and protein–protein interactions to regulate downstream outputs.

Looking ahead, the key challenge is to build a unified mechanistic framework that links ligand binding, Cu^+^ transfer, conformational dynamics, receptor clustering, and downstream protein–protein interactions. Achieving this will require multidisciplinary integration of high‐resolution structural techniques with advanced simulations capable of connecting local binding events to long‐range signaling outcomes. Such a systems‐level view will clarify how receptors achieve sensitivity, robustness, and plasticity, and how they interface with other signaling pathways. Beyond advancing our fundamental understanding, such insights will enable translational applications ranging from fine‐tuning ethylene responses in crops to enhancing post‐harvest technologies and stress resilience.

In conclusion, ethylene receptors exemplify how small‐molecule perception is coupled to metal cofactors, conformational dynamics, and higher order organization to regulate plant physiology. Continued integration of structural, biochemical, and computational approaches will be essential to unravel their complex mode of action. These insights promise to advance both fundamental plant biology and targeted strategies for crop improvement and resilience.

## CONFLICT OF INTEREST

The authors declare no competing interests.

## Data Availability

Data sharing does not apply to this article as no datasets were generated or analyzed during the current study.

## References

[tpj70810-bib-0001] Abeles, F.B. , Morgan, P.W. & Saltveit, M.E., Jr. (1992) Ethylene in plant biology, 2nd edition. San Diego, CA: Academic Press.

[tpj70810-bib-0002] Aman, S. , Swain, S. , Dutta, E. , Abbas, S. , Li, N. , Shakeel, S.N. et al. (2025) Modulation of plant growth and development through altered ethylene binding affinity of the ethylene receptor ETR1. BMC Plant Biology, 25, 436. Available from: 10.1186/s12870-025-06469-y 40186127 PMC11971883

[tpj70810-bib-0003] Azhar, B.J. , Abbas, S. , Aman, S. , Yamburenko, M.V. , Chen, W. , Muller, L. et al. (2023) Basis for high‐affinity ethylene binding by the ethylene receptor ETR1 of Arabidopsis. Proceedings of the National Academy of Sciences of the United States of America, 120, e2215195120. Available from: 10.1073/pnas.2215195120 37253004 PMC10266040

[tpj70810-bib-0004] Bellsolell, L. , Prieto, J. , Serrano, L. & Coll, M. (1994) Magnesium binding to the bacterial chemotaxis protein CheY results in large conformational changes involving its functional surface. Journal of Molecular Biology, 238, 489–495. Available from: 10.1006/jmbi.1994.1308 8176739

[tpj70810-bib-0005] Berleth, M. , Berleth, N. , Minges, A. , Hansch, S. , Burkart, R.C. , Stork, B. et al. (2019) Molecular analysis of protein‐protein interactions in the ethylene pathway in the different ethylene receptor subfamilies. Frontiers in Plant Science, 10, 726. Available from: 10.3389/fpls.2019.00726 31231408 PMC6566107

[tpj70810-bib-0006] Binder, B.M. (2008) The ethylene receptors: complex perception for a simple gas. Plant Science, 175, 8–17. Available from: 10.1016/j.plantsci.2007.12.001

[tpj70810-bib-0007] Binder, B.M. (2020) Ethylene signaling in plants. The Journal of Biological Chemistry, 295, 7710–7725. Available from: 10.1074/jbc.rev120.010854 32332098 PMC7261785

[tpj70810-bib-0008] Binder, B.M. , Kim, H.J. , Mathews, D.E. , Hutchison, C.E. , Kieber, J.J. & Schaller, G.E. (2018) A role for two‐component signaling elements in the Arabidopsis growth recovery response to ethylene. Plant Direct, 2, e00058. Available from: 10.1002/pld3.58 31245724 PMC6508545

[tpj70810-bib-0009] Binder, B.M. , O'Malley, R. , Wang, W. , Moore, J.M. , Parks, B.M. , Spalding, E.P. et al. (2004) Arabidopsis seedling growth response and recovery to ethylene. A kinetic analysis. Plant Physiology, 136, 2913–2920. Available from: 10.1104/pp.104.050369 15466220 PMC523353

[tpj70810-bib-0010] Binder, B.M. , Rodriguez, F.I. , Bleecker, A.B. & Patterson, S.E. (2007) The effects of group 11 transition metals, including gold, on ethylene binding to the ETR1 receptor and growth of Arabidopsis thaliana. FEBS Letters, 581, 5105–5109. Available from: 10.1016/j.febslet.2007.09.057 17931631

[tpj70810-bib-0011] Bisson, M.M. & Groth, G. (2010) New insight in ethylene signaling: autokinase activity of ETR1 modulates the interaction of receptors and EIN2. Molecular Plant, 3, 882–889. Available from: 10.1093/mp/ssq036 20591837

[tpj70810-bib-0012] Bisson, M.M. & Groth, G. (2012) Cyanide is an adequate agonist of the plant hormone ethylene for studying signalling of sensor kinase ETR1 at the molecular level. Biochemical Journal, 444, 261–267. Available from: 10.1042/bj20111447 22390794

[tpj70810-bib-0013] Bisson, M.M. , Kessenbrock, M. , Muller, L. , Hofmann, A. , Schmitz, F. , Cristescu, S.M. et al. (2016) Peptides interfering with protein‐protein interactions in the ethylene signaling pathway delay tomato fruit ripening. Scientific Reports, 6, 30634. Available from: 10.1038/srep30634 27477591 PMC4967898

[tpj70810-bib-0014] Bourret, R.B. (2010) Receiver domain structure and function in response regulator proteins. Current Opinion in Microbiology, 13, 142–149. Available from: 10.1016/j.mib.2010.01.015 20211578 PMC2847656

[tpj70810-bib-0015] Burg, S.P. & Burg, E.A. (1962) Role of ethylene in fruit ripening. Plant Physiology, 37, 179–189. Available from: 10.1104/pp.37.2.179 16655629 PMC549760

[tpj70810-bib-0016] Burg, S.P. & Burg, E.A. (1967) Molecular requirements for the biological activity of ethylene. Plant Physiology, 42, 144–152. Available from: 10.1104/pp.42.1.144 16656478 PMC1086501

[tpj70810-bib-0017] Casino, P. , Rubio, V. & Marina, A. (2009) Structural insight into partner specificity and phosphoryl transfer in two‐component signal transduction. Cell, 139, 325–336. Available from: 10.1016/j.cell.2009.08.032 19800110

[tpj70810-bib-0018] Chang, C. , Kwok, S.F. , Bleecker, A.B. & Meyerowitz, E.M. (1993) Arabidopsis ethylene‐response gene ETR1: similarity of product to two‐component regulators. Science, 262, 539–544. Available from: 10.1126/science.8211181 8211181

[tpj70810-bib-0019] Chen, R. , Binder, B.M. , Garrett, W.M. , Tucker, M.L. , Chang, C. & Cooper, B. (2011) Proteomic responses in Arabidopsis thaliana seedlings treated with ethylene. Molecular BioSystems, 7, 2637–2650. Available from: 10.1039/C1MB05159H 21713283

[tpj70810-bib-0020] Chen, Y.F. , Gao, Z. , Kerris, R.J., 3rd , Wang, W. , Binder, B.M. & Schaller, G.E. (2010) Ethylene receptors function as components of high‐molecular‐mass protein complexes in Arabidopsis. PLoS One, 5, e8640. Available from: 10.1371/journal.pone.0008640 20062808 PMC2799528

[tpj70810-bib-0021] Clark, K.L. , Larsen, P.B. , Wang, X. & Chang, C. (1998) Association of the Arabidopsis CTR1 Raf‐like kinase with the ETR1 and ERS ethylene receptors. Proceedings of the National Academy of Sciences of the United States of America, 95, 5401–5406. Available from: 10.1073/pnas.95.9.5401 9560288 PMC20273

[tpj70810-bib-0022] Corbineau, F. , Xia, Q. , Bailly, C. & El‐Maarouf‐Bouteau, H. (2014) Ethylene, a key factor in the regulation of seed dormancy. Frontiers in Plant Science, 5, 539. Available from: 10.3389/fpls.2014.00539 25346747 PMC4193209

[tpj70810-bib-0023] Cutsail, G., 3rd , Schott‐Verdugo, S. , Muller, L. , Debeer, S. , Groth, G. & Gohlke, H. (2022) Spectroscopic and QM/MM studies of the Cu(I) binding site of the plant ethylene receptor ETR1. Biophysical Journal, 121, 3862–3873. Available from: 10.1016/j.bpj.2022.09.007 36086818 PMC9674993

[tpj70810-bib-0024] Dluhosch, D. , Kersten, L.S. , Minges, A. , Schott‐Verdugo, S. , Gohlke, H. & Groth, G. (2025) Molecular mechanism and structural models of protein‐mediated copper transfer to the Arabidopsis thaliana ethylene receptor ETR1 at the ER membrane. Scientific Reports, 15, 38501. Available from: 10.1038/s41598-025-19915-6 41188297 PMC12586476

[tpj70810-bib-0025] Dluhosch, D. , Kersten, L.S. , Schott‐Verdugo, S. , Hoppen, C. , Schwarten, M. , Willbold, D. et al. (2024) Structure and dimerization properties of the plant‐specific copper chaperone CCH. Scientific Reports, 14, 19099. Available from: 10.1038/s41598-024-69532-y 39154065 PMC11330527

[tpj70810-bib-0026] Dong, C.H. , Rivarola, M. , Resnick, J.S. , Maggin, B.D. & Chang, C. (2008) Subcellular co‐localization of Arabidopsis RTE1 and ETR1 supports a regulatory role for RTE1 in ETR1 ethylene signaling. The Plant Journal, 53, 275–286. Available from: 10.1111/j.1365-313x.2007.03339.x 17999643 PMC2194639

[tpj70810-bib-0027] Du, H. , Wu, N. , Cui, F. , You, L. , Li, X. & Xiong, L. (2014) A homolog of ETHYLENE OVERPRODUCER, OsETOL1, differentially modulates drought and submergence tolerance in rice. The Plant Journal, 78, 834–849. Available from: 10.1111/tpj.12508 24641694

[tpj70810-bib-0028] Dubois, M. , Van Den Broeck, L. & Inze, D. (2018) The pivotal role of ethylene in plant growth. Trends in Plant Science, 23, 311–323. Available from: 10.1016/j.tplants.2018.01.003 29428350 PMC5890734

[tpj70810-bib-0029] Etchells, J.P. , Provost, C.M. & Turner, S.R. (2012) Plant vascular cell division is maintained by an interaction between PXY and ethylene signalling. PLoS Genetics, 8, e1002997. Available from: 10.1371/journal.pgen.1002997 23166504 PMC3499249

[tpj70810-bib-0030] Feng, G. , Sanderson, B.J. , Keefover‐Ring, K. , Liu, J. , Ma, T. , Yin, T. et al. (2020) Pathways to sex determination in plants: how many roads lead to Rome? Current Opinion in Plant Biology, 54, 61–68. Available from: 10.1016/j.pbi.2020.01.004 32106015

[tpj70810-bib-0031] Gallie, D.R. (2015) Ethylene receptors in plants – why so much complexity? F1000Prime Reports, 7, 39. Available from: 10.12703/P7-39 26171216 PMC4479046

[tpj70810-bib-0032] Gamble, R.L. , Coonfield, M.L. & Schaller, G.E. (1998) Histidine kinase activity of the ETR1 ethylene receptor from Arabidopsis. Proceedings of the National Academy of Sciences of the United States of America, 95, 7825–7829. Available from: 10.1073/pnas.95.13.7825 9636235 PMC22771

[tpj70810-bib-0033] Gao, Z. , Wen, C.K. , Binder, B.M. , Chen, Y.F. , Chang, J. , Chiang, Y.H. et al. (2008) Heteromeric interactions among ethylene receptors mediate signaling in Arabidopsis. The Journal of Biological Chemistry, 283, 23801–23810. Available from: 10.1074/jbc.m800641200 18577522 PMC2527101

[tpj70810-bib-0034] Gohlke, H. , Kiel, C. & Case, D.A. (2003) Insights into protein‐protein binding by binding free energy calculation and free energy decomposition for the Ras‐Raf and Ras‐RalGDS complexes. Journal of Molecular Biology, 330, 891–913. Available from: 10.1016/s0022-2836(03)00610-7 12850155

[tpj70810-bib-0035] Grefen, C. , Stadele, K. , Ruzicka, K. , Obrdlik, P. , Harter, K. & Horak, J. (2008) Subcellular localization and in vivo interactions of the Arabidopsis thaliana ethylene receptor family members. Molecular Plant, 1, 308–320. Available from: 10.1093/mp/ssm015 19825542

[tpj70810-bib-0036] Guan, R. , Su, J. , Meng, X. , Li, S. , Liu, Y. , Xu, J. et al. (2015) Multilayered regulation of ethylene induction plays a positive role in Arabidopsis resistance against pseudomonas syringae. Plant Physiology, 169, 299–312. Available from: 10.1104/pp.15.00659 26265775 PMC4577408

[tpj70810-bib-0037] Hall, A.E. , Findell, J.L. , Schaller, G.E. , Sisler, E.C. & Bleecker, A.B. (2000) Ethylene perception by the ERS1 protein in Arabidopsis. Plant Physiology, 123, 1449–1458. Available from: 10.1104/pp.123.4.1449 10938361 PMC59101

[tpj70810-bib-0038] Hall, A.E. , Grace Chen, Q. , Findell, J.L. , Eric Schaller, G. & Bleecker, A.B. (1999) The relationship between ethylene binding and dominant insensitivity conferred by mutant forms of the ETR1 ethylene receptor. Plant Physiology, 121, 291–300. Available from: 10.1104/pp.121.1.291 10482685 PMC59379

[tpj70810-bib-0039] Hall, B.P. , Shakeel, S.N. , Amir, M. , Ul Haq, N. , Qu, X. & Schaller, G.E. (2012) Histidine kinase activity of the ethylene receptor ETR1 facilitates the ethylene response in Arabidopsis. Plant Physiology, 159, 682–695. Available from: 10.1104/pp.112.196790 22467798 PMC3375934

[tpj70810-bib-0040] Hirayama, T. , Kieber, J.J. , Hirayama, N. , Kogan, M. , Guzman, P. , Nourizadeh, S. et al. (1999) RESPONSIVE‐TO‐ANTAGONIST1, a Menkes/Wilson disease‐related copper transporter, is required for ethylene signaling in Arabidopsis. Cell, 97, 383–393. Available from: 10.1016/s0092-8674(00)80747-3 10319818

[tpj70810-bib-0041] Hoppen, C. , Muller, L. , Hansch, S. , Uzun, B. , Milic, D. , Meyer, A.J. et al. (2019) Soluble and membrane‐bound protein carrier mediate direct copper transport to the ethylene receptor family. Scientific Reports, 9, 10715. Available from: 10.1038/s41598-019-47185-6 31341214 PMC6656775

[tpj70810-bib-0042] Hua, J. & Meyerowitz, E.M. (1998) Ethylene responses are negatively regulated by a receptor gene family in Arabidopsis thaliana. Cell, 94, 261–271. Available from: 10.1016/s0092-8674(00)81425-7 9695954

[tpj70810-bib-0043] Hua, J. , Sakai, H. , Nourizadeh, S. , Chen, Q.G. , Bleecker, A.B. , Ecker, J.R. et al. (1998) EIN4 and ERS2 are members of the putative ethylene receptor gene family in Arabidopsis. Plant Cell, 10, 1321–1332. Available from: 10.1105/tpc.10.8.1321 9707532 PMC144061

[tpj70810-bib-0044] Hung, Y.L. , Jiang, I. , Lee, Y.Z. , Wen, C.K. & Sue, S.C. (2016) NMR study reveals the receiver domain of Arabidopsis ETHYLENE RESPONSE1 ethylene receptor as an atypical type response regulator. PLoS One, 11, e0160598. Available from: 10.1371/journal.pone.0160598 27486797 PMC4972365

[tpj70810-bib-0045] Ju, C. , Yoon, G.M. , Shemansky, J.M. , Lin, D.Y. , Ying, Z.I. , Chang, J. et al. (2012) CTR1 phosphorylates the central regulator EIN2 to control ethylene hormone signaling from the ER membrane to the nucleus in Arabidopsis. Proceedings of the National Academy of Sciences of the United States of America, 109, 19486–19491. Available from: 10.1073/pnas.1214848109 23132950 PMC3511113

[tpj70810-bib-0046] Jumper, J. , Evans, R. , Pritzel, A. , Green, T. , Figurnov, M. , Ronneberger, O. et al. (2021) Highly accurate protein structure prediction with AlphaFold. Nature, 596, 583–589. Available from: 10.1038/s41586-021-03819-2 34265844 PMC8371605

[tpj70810-bib-0047] Kessenbrock, M. , Klein, S.M. , Muller, L. , Hunsche, M. , Noga, G. & Groth, G. (2017) Novel protein‐protein inhibitor based approach to control plant ethylene responses: synthetic peptides for ripening control. Frontiers in Plant Science, 8, 1528. Available from: 10.3389/fpls.2017.01528 28928762 PMC5591945

[tpj70810-bib-0048] Kugele, A. , Uzun, B. , Müller, L. , Schott‐Verdugo, S. , Gohlke, H. , Groth, G. et al. (2022) Mapping the helix arrangement of the reconstituted ETR1 ethylene receptor transmembrane domain by EPR spectroscopy. RSC Advances, 12, 7352–7356. Available from: 10.1039/D2RA00604A 35424698 PMC8982231

[tpj70810-bib-0049] Lemke, M. , Lakomek, N.A. & Groth, G. (2025) Structural dynamics of the plant hormone receptor ETR1 in a native‐like membrane environment. FEBS Letters, 599, 3381–3391. Available from: 10.1002/1873-3468.70153 40890953 PMC12643061

[tpj70810-bib-0050] Lemke, M. , Reiners, J. , Smits, S.H.J. , Lakomek, N. & Groth, G. (2023) Functional reconstitution and structural characterization of the plant hormone receptor ETR1 in lipid nanodiscs. Chemical Communications, 59, 9344–9347. Available from: 10.1039/D3CC02619A 37435887

[tpj70810-bib-0051] Liu, M. , Pirrello, J. , Chervin, C. , Roustan, J.P. & Bouzayen, M. (2015) Ethylene control of fruit ripening: revisiting the complex network of transcriptional regulation. Plant Physiology, 169, 2380–2390. Available from: 10.1104/pp.15.01361 26511917 PMC4677914

[tpj70810-bib-0052] Mattoo, A.K. (1991) The plant hormone ethylene. Boca Raton, FL: CRC Press.

[tpj70810-bib-0053] Mayerhofer, H. , Panneerselvam, S. , Kaljunen, H. , Tuukkanen, A. , Mertens, H.D. & Mueller‐Dieckmann, J. (2015) Structural model of the cytosolic domain of the plant ethylene receptor 1 (ETR1). The Journal of Biological Chemistry, 290, 2644–2658. Available from: 10.1074/jbc.M114.587667 25451923 PMC4317023

[tpj70810-bib-0054] Milic, D. , Dick, M. , Mulnaes, D. , Pfleger, C. , Kinnen, A. , Gohlke, H. et al. (2018) Recognition motif and mechanism of ripening inhibitory peptides in plant hormone receptor ETR1. Scientific Reports, 8, 3890. Available from: 10.1038/s41598-018-21952-3 29497085 PMC5832771

[tpj70810-bib-0055] Mira‐Rodado, V. , Veerabagu, M. , Witthoft, J. , Teply, J. , Harter, K. & Desikan, R. (2012) Identification of two‐component system elements downstream of AHK5 in the stomatal closure response of Arabidopsis thaliana. Plant Signaling & Behavior, 7, 1467–1476. Available from: 10.4161/psb.21898 22951399 PMC3548872

[tpj70810-bib-0056] Moretti, C.L. , Mattos, L.M. , Calbo, A.G. & Sargent, S.A. (2010) Climate changes and potential impacts on postharvest quality of fruit and vegetable crops: a review. Food Research International, 43, 1824–1832. Available from: 10.1016/j.foodres.2009.10.013

[tpj70810-bib-0057] Mudge, A.J. , Mehdi, S. , Michaels, W. , Orosa‐Puente, B. , Shen, W. , Tomlinson, C. et al. (2025) POLARIS is a copper‐binding peptide that interacts with ETR1 to negatively regulate ethylene signaling in Arabidopsis. Plant Communications, 6, 101432. Available from: 10.1016/j.xplc.2025.101432 40574333 PMC12744750

[tpj70810-bib-0058] Müller‐Dieckmann, H.‐J. , Grantz, A.A. & Kim, S.‐H. (1999) The structure of the signal receiver domain of the Arabidopsis thaliana ethylene receptor ETR1. Structure, 7, 1547–1556. Available from: 10.1016/s0969-2126(00)88345-8 10647185

[tpj70810-bib-0059] Nemhauser, J.L. , Hong, F. & Chory, J. (2006) Different plant hormones regulate similar processes through largely nonoverlapping transcriptional responses. Cell, 126, 467–475. Available from: 10.1016/j.cell.2006.05.050 16901781

[tpj70810-bib-0060] O'Malley, R.C. , Rodriguez, F.I. , Esch, J.J. , Binder, B.M. , O'donnell, P. , Klee, H.J. et al. (2005) Ethylene‐binding activity, gene expression levels, and receptor system output for ethylene receptor family members from Arabidopsis and tomato. The Plant Journal, 41, 651–659. Available from: 10.1111/j.1365-313x.2004.02331.x 15703053

[tpj70810-bib-0061] Perata, P. (2020) Ethylene signaling controls fast oxygen sensing in plants. Trends in Plant Science, 25, 3–6. Available from: 10.1016/j.tplants.2019.10.010 31734094

[tpj70810-bib-0062] Pfleger, C. , Minges, A. , Boehm, M. , Mcclendon, C.L. , Torella, R. & Gohlke, H. (2017) Ensemble‐ and rigidity theory‐based perturbation approach to analyze dynamic Allostery. Journal of Chemical Theory and Computation, 13, 6343–6357. Available from: 10.1021/acs.jctc.7b00529 29112408

[tpj70810-bib-0063] Pirrung, M.C. , Bleecker, A.B. , Inoue, Y. , Rodriguez, F.I. , Sugawara, N. , Wada, T. et al. (2008) Ethylene receptor antagonists: strained alkenes are necessary but not sufficient. Chemistry & Biology, 15, 313–321. Available from: 10.1016/j.chembiol.2008.02.018 18420138

[tpj70810-bib-0064] Pufahl, R.A. , Singer, C.P. , Peariso, K.L. , Lin, S.J. , Schmidt, P.J. , Fahrni, C.J. et al. (1997) Metal ion chaperone function of the soluble Cu(I) receptor Atx1. Science, 278, 853–856. Available from: 10.1126/science.278.5339.853 9346482

[tpj70810-bib-0065] Qiao, H. , Shen, Z. , Huang, S.S. , Schmitz, R.J. , Urich, M.A. , Briggs, S.P. et al. (2012) Processing and subcellular trafficking of ER‐tethered EIN2 control response to ethylene gas. Science, 338, 390–393. Available from: 10.1126/science.1225974 22936567 PMC3523706

[tpj70810-bib-0066] Rodriguez, F.I. , Esch, J.J. , Hall, A.E. , Binder, B.M. , Schaller, G.E. & Bleecker, A.B. (1999) A copper cofactor for the ethylene receptor ETR1 from Arabidopsis. Science, 283, 996–998. Available from: 10.1126/science.283.5404.996 9974395

[tpj70810-bib-0067] Rüffer, B. , Thielmann, Y. , Lemke, M. , Minges, A. & Groth, G. (2024) Crystallization of ethylene plant hormone receptor‐screening for structure. Biomolecules, 14, 375. Available from: 10.3390/biom14030375 38540793 PMC10968091

[tpj70810-bib-0068] Sakai, H. , Hua, J. , Chen, Q.G. , Chang, C. , Medrano, L.J. , Bleecker, A.B. et al. (1998) ETR2 is an ETR1‐like gene involved in ethylene signaling in Arabidopsis. Proceedings of the National Academy of Sciences of the United States of America, 95, 5812–5817. Available from: 10.1073/pnas.95.10.5812 9576967 PMC20462

[tpj70810-bib-0069] Schaller, G.E. , Ladd, A.N. , Lanahan, M.B. , Spanbauer, J.M. & Bleecker, A.B. (1995) The ethylene response mediator ETR1 from Arabidopsis forms a disulfide‐linked dimer. The Journal of Biological Chemistry, 270, 12526–12530. Available from: 10.1074/jbc.270.21.12526 7759498

[tpj70810-bib-0070] Scharein, B. , Voet‐Van‐Vormizeele, J. , Harter, K. & Groth, G. (2008) Ethylene signaling: identification of a putative ETR1‐AHP1 phosphorelay complex by fluorescence spectroscopy. Analytical Biochemistry, 377, 72–76. Available from: 10.1016/j.ab.2008.03.015 18384742

[tpj70810-bib-0071] Schott‐Verdugo, S. , Muller, L. , Classen, E. , Gohlke, H. & Groth, G. (2019) Structural model of the ETR1 ethylene receptor transmembrane sensor domain. Scientific Reports, 9, 8869. Available from: 10.1038/s41598-019-45189-w 31222090 PMC6586836

[tpj70810-bib-0072] Sisler, E.C. (1977) Ethylene activity of some pi acceptor compounds. Tobacco Science, 21, 43–45.

[tpj70810-bib-0073] Sisler, E.C. , Blankenship, S.M. & Guest, M. (1990) Competition of cyclooctenes and cyclooctadienes for ethylene binding and activity in plants. Plant Growth Regulation, 9, 157–164. Available from: 10.1007/BF00027443

[tpj70810-bib-0074] Sisler, E.C. , Serek, M. & Dupille, E. (1996) Comparison of cyclopropene, 1‐methylcyclopropene, and 3, 3‐dimethylcyclopropene as ethylene antagonists in plants. Plant Growth Regulation, 18, 169–174. Available from: 10.1007/BF00024378

[tpj70810-bib-0075] Stock, A.M. , Martinez‐Hackert, E. , Rasmussen, B.F. , West, A.H. , Stock, J.B. , Ringe, D. et al. (1993) Structure of the Mg(2+)‐bound form of CheY and mechanism of phosphoryl transfer in bacterial chemotaxis. Biochemistry, 32, 13375–13380. Available from: 10.1021/bi00212a001 8257674

[tpj70810-bib-0076] Street, I.H. , Aman, S. , Zubo, Y. , Ramzan, A. , Wang, X. , Shakeel, S.N. et al. (2015) Ethylene inhibits cell proliferation of the Arabidopsis root meristem. Plant Physiology, 169, 338–350. Available from: 10.1104/pp.15.00415 26149574 PMC4577392

[tpj70810-bib-0077] Tao, J.J. , Chen, H.W. , Ma, B. , Zhang, W.K. , Chen, S.Y. & Zhang, J.S. (2015) The role of ethylene in plants under salinity stress. Frontiers in Plant Science, 6, 1059. Available from: 10.3389/fpls.2015.01059 26640476 PMC4661241

[tpj70810-bib-0078] Thomashow, M.F. (2010) Molecular basis of plant cold acclimation: insights gained from studying the CBF cold response pathway. Plant Physiology, 154, 571–577. Available from: 10.1104/pp.110.161794 20921187 PMC2948992

[tpj70810-bib-0079] Voet‐Van‐Vormizeele, J. & Groth, G. (2008) Ethylene controls autophosphorylation of the histidine kinase domain in ethylene receptor ETR1. Molecular Plant, 1, 380–387. Available from: 10.1093/mp/ssn004 19825547

[tpj70810-bib-0080] Wang, W. , Esch, J.J. , Shiu, S.H. , Agula, H. , Binder, B.M. , Chang, C. et al. (2006) Identification of important regions for ethylene binding and signaling in the transmembrane domain of the ETR1 ethylene receptor of Arabidopsis. Plant Cell, 18, 3429–3442. Available from: 10.1105/tpc.106.044537 17189345 PMC1785413

[tpj70810-bib-0081] Wang, W. , Hall, A.E. , O'Malley, R. & Bleecker, A.B. (2003) Canonical histidine kinase activity of the transmitter domain of the ETR1 ethylene receptor from Arabidopsis is not required for signal transmission. Proceedings of the National Academy of Sciences of the United States of America, 100, 352–357. Available from: 10.1073/pnas.0237085100 12509505 PMC140975

[tpj70810-bib-0082] Wei, S. , Yang, Y. , Yuan, Y. , Du, L. , Xue, H. & Ouyang, B. (2022) NMR detection and structural modeling of the ethylene receptor LeETR2 from tomato. Membranes, 12, 107. Available from: 10.3390/membranes12020107 35207029 PMC8879215

[tpj70810-bib-0083] Wen, X. , Zhang, C. , Ji, Y. , Zhao, Q. , He, W. , An, F. et al. (2012) Activation of ethylene signaling is mediated by nuclear translocation of the cleaved EIN2 carboxyl terminus. Cell Research, 22, 1613–1616. Available from: 10.1038/cr.2012.145 23070300 PMC3494400

[tpj70810-bib-0084] Wuriyanghan, H. , Zhang, B. , Cao, W.H. , Ma, B. , Lei, G. , Liu, Y.F. et al. (2009) The ethylene receptor ETR2 delays floral transition and affects starch accumulation in rice. Plant Cell, 21, 1473–1494. Available from: 10.1105/tpc.108.065391 19417056 PMC2700534

[tpj70810-bib-0085] Zdarska, M. , Cuyacot, A.R. , Tarr, P.T. , Yamoune, A. , Szmitkowska, A. , Hrdinova, V. et al. (2019) ETR1 integrates response to ethylene and cytokinins into a single multistep phosphorelay pathway to control root growth. Molecular Plant, 12, 1338–1352. Available from: 10.1016/j.molp.2019.05.012 31176773 PMC8040967

[tpj70810-bib-0086] Zhao, H. , Duan, K.X. , Ma, B. , Yin, C.C. , Hu, Y. , Tao, J.J. et al. (2020) Histidine kinase MHZ1/OsHK1 interacts with ethylene receptors to regulate root growth in rice. Nature Communications, 11, 518. Available from: 10.1038/s41467-020-14313-0 PMC6981129 31980616

[tpj70810-bib-0087] Zimmermann, M. , Clarke, O. , Gulbis, J.M. , Keizer, D.W. , Jarvis, R.S. , Cobbett, C.S. et al. (2009) Metal binding affinities of Arabidopsis zinc and copper transporters: selectivities match the relative, but not the absolute, affinities of their amino‐terminal domains. Biochemistry, 48, 11640–11654. Available from: 10.1021/bi901573b 19883117

